# Pre-transplant whole blood transcriptomic profiling identifies mRNAs linked to cirrhosis-associated immune dysfunction as candidate predictors of clinical outcomes after liver transplantation: an exploratory study

**DOI:** 10.3389/ti.2026.16292

**Published:** 2026-08-28

**Authors:** Marie-Charlotte Delignette, Estelle Peronnet, Arnaud Riff, Teresa Antonini, Maxime Bodinier, Elisabeth Cerrato, Solène Pantel, Elsa Coz, Xavier Muller, Guillaume Rossignol, Jean-Yves Mabrut, Jérôme Dumortier, Céline Guichon, Alice Blet, Frederic Aubrun, Guillaume Monneret, Fanny Lebossé

**Affiliations:** 1 Anesthesiology and Intensive Care Department, Croix-Rousse Hospital, Lyon Liver Institute, Hospices Civils of Lyon, Lyon, France; 2 Everest Lyon Liver Institute, Lyon, France; 3 EA 7426 Pathophysiology of Injury-Induced Immunosuppression (PI3), Lyon 1 University, Hospices Civils of Lyon, bioMérieux, Edouard Herriot Hospital, Lyon, France; 4 Université Claude Bernard Lyon 1, Villeurbanne, France; 5 Hepatology Department, Croix-Rousse Hospital, Lyon Liver Institute, Hospices Civils of Lyon, Lyon, France; 6 INSERM UMR 1350 - PaThLiv, Lyon, France; 7 Clinical Research Center, Croix-Rousse Hospital, Lyon Liver Institute, Hospices Civils of Lyon, Lyon, France; 8 Liver Transplantation Department, Croix-Rousse Hospital, Lyon Liver Institute, Hospices Civils of Lyon, Lyon, France; 9 Hepatogastroenterology Department, Edouard Herriot Hospital, Hospices Civils of Lyon, Lyon, France; 10 Research on Healthcare Performance (RESHAPE), INSERM U1290, Lyon, France; 11 Immunology Department, Edouard Herriot Hospital, Lyon Liver Institute, Hospices Civils of Lyon, Lyon, France

**Keywords:** liver transplantation, cirrhosis-associated immune dysfunction, transcriptomics, immune monitoring, infections

## Abstract

Cirrhosis-associated immune dysfunction (CAID) contributes to poor outcomes after liver transplantation (LT), but pre-transplant immune predictors remain insufficiently defined. We investigated whether pre-transplant whole blood transcriptomic profiling was associated with post-LT outcomes in this exploratory ancillary study of the prospective EDMONHG cohort. Transcriptomic analysis of 26 immune-related genes was performed on 97 LT recipients. PCA and volcano plots identified candidate biomarkers; associations with outcomes were assessed using ROC analyses and Kaplan-Meier estimates with cohort medians as thresholds. PC1 (43.5% of variance) decreased progressively from cACLD to ALF (p < 0.001) and was lower in post-LT infected patients (p = 0.044). Within the first month, 34 patients (35%) developed infections; 7 (7.2%) died within 1 year. Three genes met predefined exploratory criteria (q < 0.10, AUC > 0.65) for infections: GNLY and IL7R were downregulated and IL10 upregulated. Low GNLY or high IL10 expression was associated with reduced 30-day infection-free survival (p = 0.019 and p = 0.013). CIITA, IL10, CD177 and S100A9 showed associations with one-year survival, though results should be treated as exploratory given the limited number of events. Pre-transplant transcriptomics captures CAID severity and identifies candidate gene signatures associated with post-LT outcomes. These hypothesis-generating findings warrant validation in larger multicenter cohorts.

## Graphical Abstract

**Figure:**
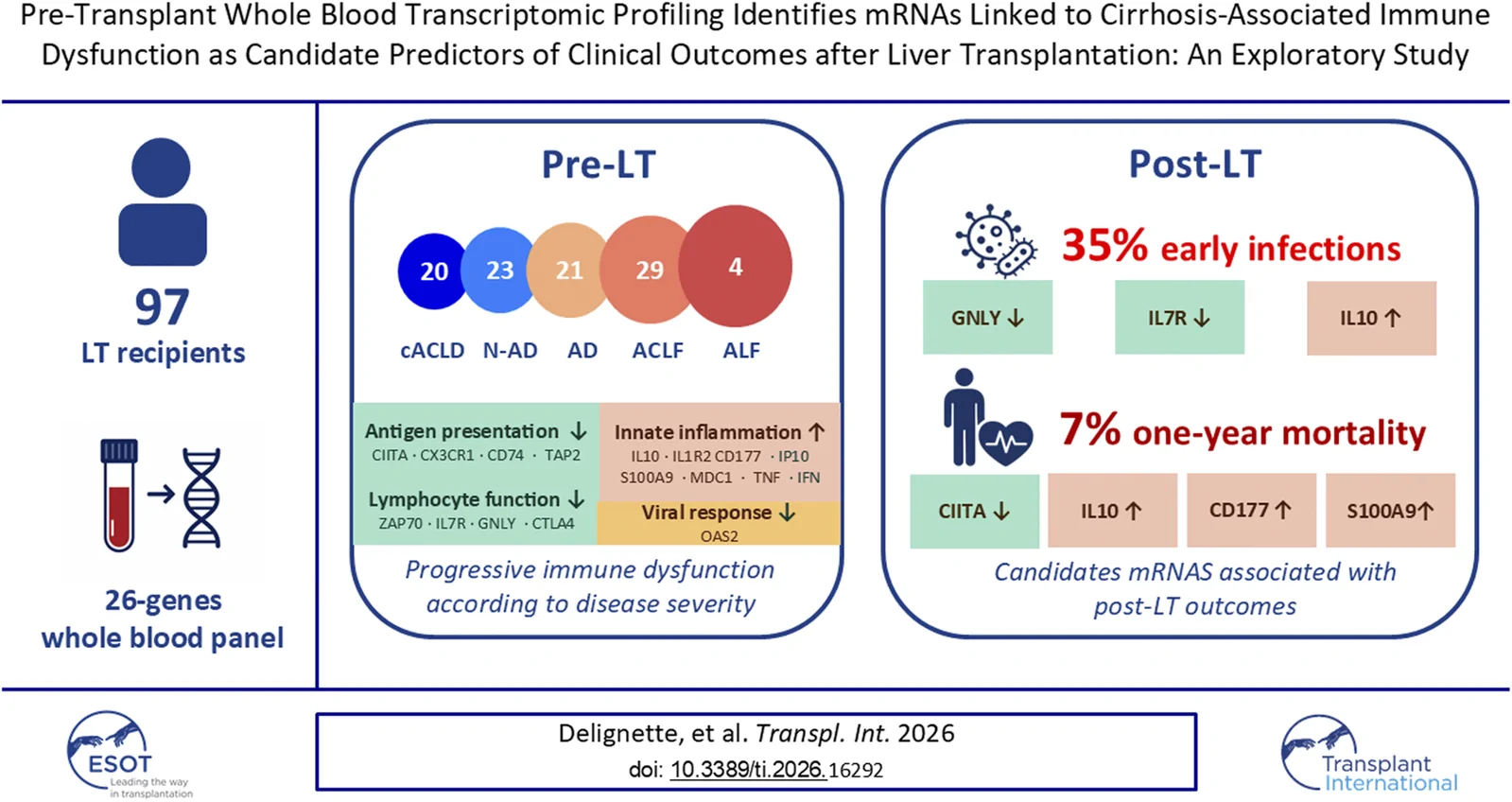

## Introduction

Cirrhosis-associated immune dysfunction (CAID) is characterized by persistent systemic inflammation and progressive immune suppression, significantly contributing to increased morbidity and mortality in patients with cirrhosis [1]. Patients with Acute-on-Chronic Liver Failure (ACLF), who exhibit the most severe immune dysfunction, experience higher rates of infection and elevated mortality before liver transplantation (LT) [2, 3]. This higher risk of mortality and infection persists after LT compared with patients without ACLF [4]. The persistence of an excessive risk of death despite LT could suggest a potential link between pre-LT immune dysfunction and poor outcomes following LT. Several studies have reported associations between pre-transplant immune status and post-LT outcomes, including sepsis and mortality, using protein-level or cellular immune markers [5–7]. However, the relationship between pre-transplant immune dysfunction and its evolution or impact on post-transplant outcomes remains understudied and there are currently no tools available for immune monitoring in the context of LT.

Recently, transcriptomic studies have contributed to decipher the mechanisms of CAID and systemic inflammation in end-stage liver disease. Weiss et al. characterized CAID features, notably neutrophil alterations, using transcriptomic analysis at different stages of cirrhosis including ACLF [8]. Gene scores developed from blood RNA sequencing may be superior to clinical scores in reflecting the intensity of systemic inflammation in patients with acute decompensation of cirrhosis (AD) and ACLF [9]. Recent transcriptomic studies on sepsis have highlighted the potential of blood-based gene expression profiling to uncover immune signatures predictive of outcomes and to stratify patients based on their immune status. In particular, immune transcriptomic endotypes have been associated with differential risks of mortality and secondary infections in patients with septic shock, independently of clinical severity scores [10]. Given the close immunological parallels between septic shock and ACLF, both characterized by systemic inflammation and immune exhaustion, these findings support the relevance of transcriptomic approaches to better characterize and predict outcomes of cirrhosis-associated immune dysfunction.

We recently reported in the EdMonHG cohort that post-LT mHLA-DR trajectories were independently associated with early infections and one-year survival [11]. However, none of the immunological markers measured before LT, including mHLA-DR expression and lymphocyte counts, were associated with post-LT outcomes in that study, underscoring the need for more granular pre-LT immune characterization.

This motivated the design of an exploratory transcriptomic analysis as an ancillary study of the EdMonHG cohort, which pursued two objectives: first, to preliminarily characterize the spectrum of immune dysfunction across liver disease severity stages using a targeted 26-gene whole blood panel; and second, to explore whether specific pre-transplant transcriptomic immune signatures might be associated with post-LT outcomes, namely, early infections and one-year survival.

## Materials and methods

### Study population

This work represents an ancillary study of the prospective, single-center EdMonHG cohort (ClinicalTrials.gov identifier NCT03995537) [11]. The inclusion criteria were adult patients awaiting LT due to compensated Advanced Chronic Liver Disease (cACLD), decompensated ACLD (including acute decompensation (AD), non-acute chronic decompensation (N-AD) and acute-on-chronic liver failure (ACLF)), or acute liver failure (ALF). Exclusion criteria were ongoing immunosuppressive treatment (except corticosteroids), patients without underlying liver disease, and patients awaiting retransplantation or multiorgan transplantation.

### Recipient, donor and perioperative variables

We systematically collected recipient demographic and clinical variables (age, sex, liver disease etiology, clinical presentation stage (cACLD, N-AD, AD, ACLF, ALF), clinical severity scores routinely used to assess pre-transplant disease severity in LT candidates: Model for End-stage Liver Disease (MELD) score, Sequential Organ Failure Assessment (SOFA) score, ACLF grade and number of organ failures according to the CLIF-ACLF definition, and hospitalization status at the time of LT. We also collected donor variables (age, sex, body mass index, donation type, and cause of death), and perioperative/surgical variables (intraoperative packed red blood cell transfusion requirements, warm and cold ischemia times, and hepaticojejunostomy).

### Blood collection and transcriptomic analyses

In line with the EdMonHG biobanking protocol, immune monitoring was performed every 3 months in cACLD and N-AD patients, and during admission for AD or ACLF patients. As a result, the median interval between the pre-LT sampling and transplantation was 9 days [IQR: 1–36]. T lymphocyte count and mHLA-DR expression were assessed in parallel at the same time point, using flow cytometry as previously described in the EdMonHG cohort [11].

For transcriptomic analyses, blood samples were drawn into PAXgene tubes for optimal RNA stabilization. After 2 h at room temperature, the samples were frozen at −80 °C following the manufacturer’s recommendations. We performed targeted transcriptomic analysis using RT-qPCR on a panel of 26 genes related to inflammation and immune response, the Immune Profiling Panel (IPP prototype, bioMérieux, Marcy l’Etoile, France). Of note, these 26 genes were selected based on previous work [10, 12]. This panel covered genes coding for soluble markers of the inflammatory response (pro- and anti-inflammatory), membrane markers associated with innate and adaptive responses, alarmins and genes involved in the regulation of cell cycle or metabolism (Supplementary Figure 1). The mRNA expression level was determined using the FilmArray Torch System (bioMérieux) by injecting 100 µL of the PAXgene whole blood into the IPP prototype pouch. The gene expression data were normalized using reference housekeeping genes from the original publication detailing the approach [13]. The selection of these reference genes was based on previous studies conducted by our team and other groups [14–16].

### Post liver-transplantation outcomes

The primary outcomes were the occurrence of significant early post-LT infections (within the first month following LT) and one-year survival post-LT. Post-operative infections were defined according to the American Society of Transplantation [17] (see definitions in Supplementary Materials) and only significant infections were recorded (excluding uncomplicated cystitis, colitis and catheter colonization). Two independent experts confirmed the cases of post-LT infections *a posteriori*, before performing transcriptomic assays.

Post-LT outcome variables, including early allograft dysfunction, biliary and vascular complications, and prolonged postoperative corticosteroid use, were also collected.

### Statistics

The results were expressed as medians and interquartile ranges (IQRs) or as numbers and percentages (%). All 97 patients with available pre-transplant transcriptomic data were included; there was no missing data for the primary outcomes or transcriptomic variables.

To explore the overall structure of immune dysregulation, principal component analysis (PCA) was performed on the normalized expression values of all 26 transcriptomic markers, after centering and scaling each gene to unit variance (i.e., PCA was computed on the correlation rather than the covariance matrix), so that no individual gene’s raw variance could disproportionately drive the principal components. PCA scores were compared across disease severity stages and according to post-LT outcomes using the Kruskal-Wallis and Wilcoxon rank-sum tests, respectively. Gene loadings on PC1 were examined to identify the main contributors to immune variance. Candidate biomarkers were identified through a two-step approach. For each gene included in the transcriptomic panel, normalized expression levels were compared between groups using Wilcoxon rank-sum tests for binary outcomes (post-LT infection and one-year survival) and Kruskal–Wallis tests for comparisons across liver disease severity stages and liver disease etiologies. To account for multiple testing, p-values were adjusted using the Benjamini–Hochberg false discovery rate (FDR) procedure, and both nominal p-values and adjusted q-values were reported. In parallel, receiver operating characteristic (ROC) analyses were performed and areas under the curve (AUCs) with 95% confidence intervals were calculated for each marker. Volcano plots summarized these analyses by representing the median difference in normalized expression on the x-axis and the −log10(p-value) on the y-axis, with dot size proportional to the AUC. Genes fulfilling both q < 0.10 and AUC > 0.65 criteria were considered candidate biomarkers, highlighted in color on volcano plots, and selected for further analyses including boxplots of normalized expression, threshold-based survival analyses using Kaplan–Meier estimates with cohort median cut-offs, and calibration analyses. An AUC threshold of 0.65 was selected to enrich for markers with at least moderate discriminative ability in this exploratory study. Differences between groups in survival analyses were assessed using the log-rank test. Calibration plots for individual logistic regression models were generated to evaluate the predictive performance of candidate biomarkers. Calibration was assessed using the Hosmer–Lemeshow goodness-of-fit test and generalized additive model (GAM) smoothing, in order to assess agreement between predicted probabilities and observed outcome rates.

Pre-transplant mHLA-DR expression and lymphocyte counts were compared across disease severity stages alongside transcriptomic markers, using the Kruskal-Wallis test, for contextual reference. As their associations with post-LT outcomes have been previously reported in this cohort [11], these markers were not included in the outcome analyses. Given that this was a single-center study with a limited number of outcome events, particularly for one-year mortality (n = 7), the analysis remained exploratory and no formal multivariable analysis could be performed.

R version 4.0.2 (R Core Team 2020, Vienna, Austria) and GraphPad Prism 6.0 (GraphPad Software, La Jolla California, USA) were used for all analyses. The significance level was set at p < 0.05 or q < 0.10 for the Benjamini-Hochberg test.

### Guidelines

This study was conducted in accordance with the principles of the Declaration of Helsinki and approved by Institutional Review Board (Comité de Protection des Personnes Ile de France XI, approval number 19039-40433). Written informed consent was obtained from all participants prior to enrollment. This study adhered to the Strengthening the Reporting of Observational Studies in Epidemiology (STROBE) reporting guidelines. This study was registered in the ClinicalTrials.gov registry (NCT03995537, date: June 20, 2019).

## Results

### Clinical characteristics

A total of 130 patients were initially included in the EdMonHG cohort between February 2020 and May 2023. Among these patients, 99 underwent liver transplantation (LT), and 97 of these patients had available transcriptomic data and were therefore included in the current ancillary study.

LT recipients were predominantly male (n = 78, 80%), with a median age of 56 years [48–61]. At pre-LT, 73 patients had decompensated ACLD (23 with N-AD, 21 with AD, and 29 with ACLF), 20 patients had cACLD, and 4 patients were admitted for ALF. The most common underlying liver disease among ACLD patients was alcohol-related liver disease (ALD, n = 68, 73%), followed by viral infection (n = 11, 12% - see details in Supplementary Materials), autoimmunity (n = 8, 9%), metabolic dysfunction-associated steatohepatitis (MASH) (n = 3, 3%), Wilson disease (n = 2, 2%), and progressive familial intrahepatic cholestasis (n = 1, 1%). The etiologies of ALF were acute HBV infection (n = 2), autoimmune hepatitis (n = 1) and posttraumatic ischemia (n = 1). Only three patients received pre-transplant corticosteroids. Among the ACLF patients, the median number of organ failures (OF) pre-LT was 2 [1–3], 7 (24%) patients presented with grade 1 ACLF, 16 (55%) with grade 2 ACLF and 6 patients (21%) with grade 3 ACLF. Coagulation failure (20/29), liver failure (19/29), and hemodynamic failure (10/29) were the most common organ failures. The median MELD score in the entire cohort pre-LT was 20 [14–28] and the median interval between pre-LT immune monitoring and LT was 9 days [1–36]. At the time of LT, 20 patients (20.6%) were hospitalized in the ICU, 12 (12.4%) in a hepatology ward, and 65 (67.0%) were outpatients.

Regarding donor and graft characteristics, donors were predominantly male (n = 62, 64%) with a median age of 66 years [51–72] and a median BMI of 25 kg/m^2^ [23–28]. Causes of donor death were cerebrovascular (n = 48, 49%), traumatic (n = 17, 18%), anoxic (n = 30, 31%) and meningitis (n = 2, 2%). Of the total cohort, 17.5% (n = 17) were classified as Donation after Circulatory Death (DCD) donors. Median warm ischemia time was 37 min [27–51] and median cold ischemia time was 395 min [323–469]. Biliodigestive anastomosis was performed in 9 patients (9%).

Regarding perioperative and early post-transplant data, the median number of packed red blood cell units transfused intraoperatively was 2 [0–5]. Early allograft dysfunction (EAD) according to Olthoff criteria (see details of definition in Supplementary Materials) [18] occurred in 30 patients (31%). Biliary complications were observed in 14 patients (14%), vascular complications in 13 patients (13%), and prolonged corticosteroid therapy (>7 days) was required in 12 patients (12%).

### Overall transcriptomic landscape: principal component analysis

To characterize the overall structure of immune gene expression in our cohort, we first performed PCA on the normalized expression values of all 26 transcriptomic markers. The first two principal components explained 43.5% and 9.5% of total variance, respectively (Supplementary Figure 2). PC1 loading analysis identified two gene clusters with opposing contributions. Genes contributing positively to PC1 (loadings >0.75) included markers of monocyte antigen presentation (CIITA, TAP2, CX3CR1), lymphocyte signaling (ZAP70, IL7R, CD3D), cytotoxic function (GNLY) and cell cycle regulation (CCNB1IP1). Conversely, genes contributing negatively to PC1 (loadings < -0.75) were predominantly associated with innate myeloid inflammatory and immunoregulatory responses, including the decoy interleukin-1 receptor IL1R2, a marker of suppressive myeloid activation, and the alarmin S100A9*.* PC2 explained a smaller proportion of total variance and was primarily driven by genes involved in innate inflammatory regulation and immune checkpoint signaling, including CD274 (PD-L1), C3AR1, and IL1RN (loadings >0.60). These genes are associated with inflammatory feedback regulation, complement-mediated innate immune activation, and counter-regulatory anti-inflammatory pathways.

### Association between transcriptomic markers and pre-LT liver disease severity

**FIGURE 1:**
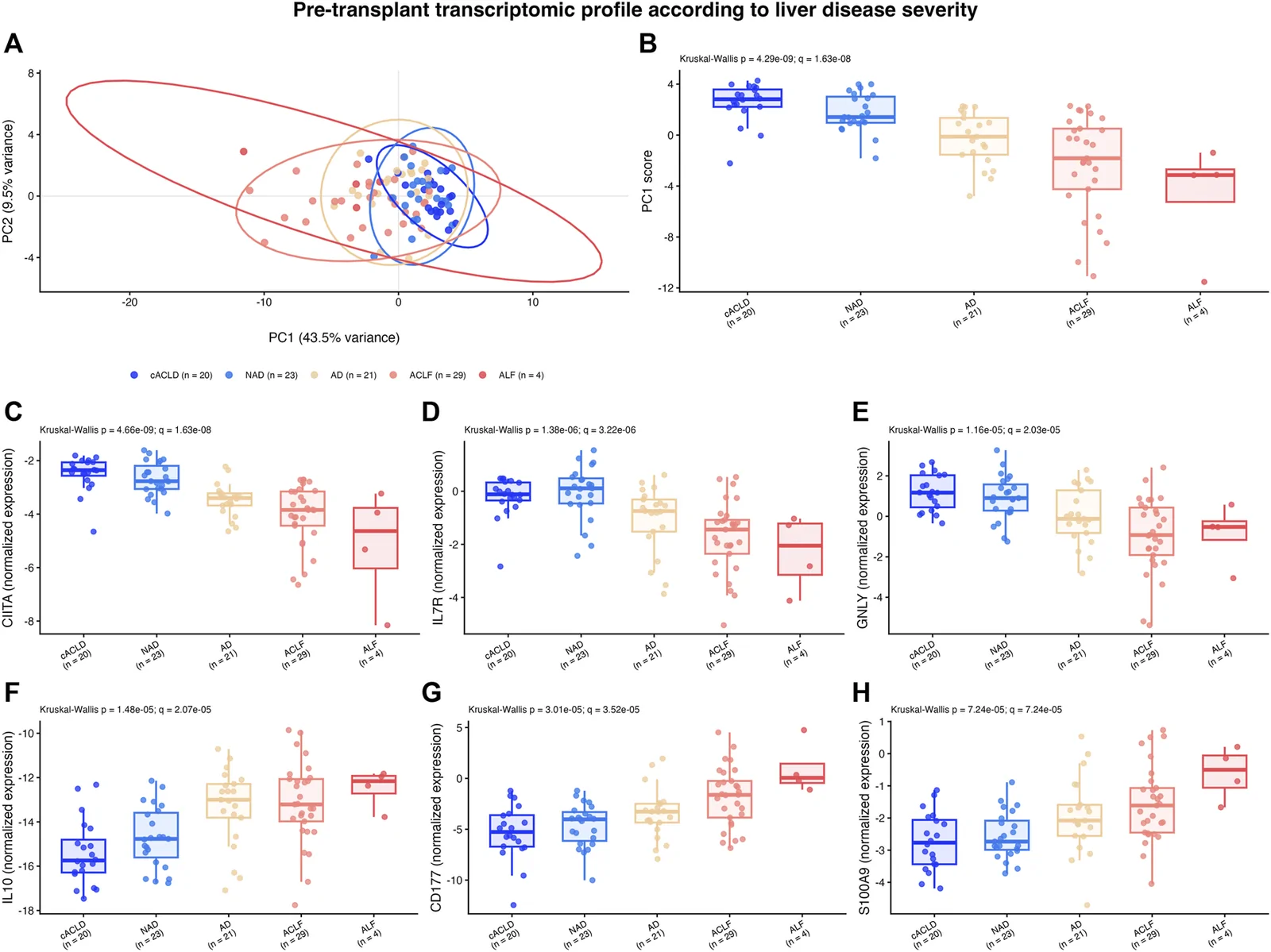
Pre-transplant transcriptomic profile according to liver disease severity. **(A)** Principal component analysis (PCA) biplot of the 26 immune-related genes, with samples colored by liver disease severity stage. Confidence ellipses are shown for each group. **(B)** PC1 score according to disease severity stage (Kruskal–Wallis p = 4.29 × 10^−9^). **(C–H)** Normalized expression of CIITA, IL7R, GNLY, IL10, CD177 and S100A9 according to disease severity stage. Nominal p-values and Benjamini–Hochberg adjusted q-values are indicated on each panel. Gene expression values were normalized to reference genes. cACLD, compensated advanced chronic liver disease; NAD, non-acute decompensation; AD, acute decompensation; ACLF, acute-on-chronic liver failure; ALF, acute liver failure.

On the PCA biplot, samples colored by disease severity showed a progressive spread along the PC1 axis from cACLD to ALF, with patients with more advanced disease shifting toward negative PC1 values (Figure 1A). PC1 score decreased progressively from cACLD to ALF (Kruskal-Wallis p < 0.0001, Figure 1B), indicating that the dominant axis of transcriptomic variability reflects the CAID severity continuum. Figures 1C–H illustrate representative genes from distinct immune functions, monocyte antigen presentation (CIITA), lymphocyte signaling (IL7R), cytotoxic function (GNLY), anti-inflammatory cytokines (IL10), neutrophil activation (CD177) and alarmin-related inflammation (S100A9), all showing significant progressive modulation across severity stages (all adjusted q < 0.001). Expression levels of all additional significantly modulated genes are shown in Supplementary Figure 3, alongside mHLA-DR expression and lymphocyte counts (q < 0.001 and q < 0.05 respectively). To assess whether the transcriptomic differences observed across severity stages could be explained by etiological heterogeneity, gene expression was compared across the three main underlying etiologies (ALD, viral, autoimmune). No significant differences were observed for any of the 26 genes after correction for multiple testing (all q > 0.10, Supplementary Table 1), although several genes reached nominal significance before adjustment. No etiology-specific transcriptomic differences could therefore be detected in this analysis; given the modest sample size, however, this exploratory comparison was not powered to formally exclude such differences.

### Association between pre-LT immune markers and post-LT infections

Among 97 patients, 34 (35%) experienced at least one significant post-LT infection within the first month. The median time to diagnosis was 9 [6–14] days post-LT. The most frequent infections were intra-abdominal infections (n = 19), followed by pneumonia (n = 15), bacteremia (n = 2), fungal infections (n = 3) and viral infections (n = 2). Microbiological details are provided in Supplementary Materials.

Regarding recipient characteristics, infected patients presented with a greater number of pre-LT organ failures (median 1 [0–2] vs. 0 [0–1], p = 0.04), and a higher rate of ICU admission at the time of LT (38% vs. 11%, p = 0.004); no significant differences were observed in MELD or SOFA scores (Table 1). Regarding donor and perioperative factors, intraoperative packed red blood cell transfusion requirements were significantly higher in infected patients (median 4 [2–8] vs. 2 [0–5] units, p = 0.005), EAD was more frequent (44.1% vs. 23.8%, p = 0.04), and a trend toward lower DCD utilization was observed (5.9% vs. 23.8%, p = 0.05, Table 1).

**TABLE 1:** Recipient’s characteristics and severity score according to the occurrence of post-LT infections.

Recipient’s characteristics	Post-LT infections n = 34	No post-LT infections n = 63	p
Age (years)	53 [45–60]	58 [50–61]	0.11
Sex (male)	30 (88.2)	48 (76.2)	0.25
Baseline severity scores			
Clinical presentation	​	​	0.06
cACLD	5 (14.7)	15 (23.8)	​
N-AD	6 (17.6)	17 (27.0)	​
AD	5 (14.7)	16 (25.4)	​
ACLF	15 (44.1)	14 (22.2)	​
ALF	3 (8.8)	1 (1.6)	​
OF (ACLF/ALF)	**18 (52.9)**	**15 (23.8)**	**0.008**
Number of organ failures	**1 [0–2]**	**0 [0–1]**	**0.04**
MELD score	22 [16–36]	20 [13–26]	0.07
SOFA score	5 [2–8]	4 [3–6]	0.11
Hospitalisation status at LT			
ICU	13 (38.2)	7 (11.1)	**0.004**
Hepatology ward	5 (14.7)	7 (11.1)	​
Outpatient	16 (47.1)	49 (77.8)	​
Donors characteristics			
Age (years)	69 [51–74]	54 [51–71]	0.41
Sex (male)	17 (50.0)	45 (71.4)	0.06
BMI (kg/m^2^)	25 [23–27]	25 [22–28]	0.98
DCD	2 (5.9)	15 (23.8)	0.05
Causes of donor death	​	​	0.12
Cerebrovascular	18 (52.9)	30 (47.6)	​
Traumatic	7 (20.6)	10 (15.9)	​
Anoxic	7 (20.6)	23 (36.5)	​
Meningitis	2 (5.9)	0 (0)	​
Surgery characteristics			
PRBC transfusion	4 [2–8]	2 [0–5]	**0.005**
Warm ischemia	37 [29–40]	37 [25–41]	0.73
Cold ischemia	398 [330–466]	393 [324–470]	0.71
Hepaticojejunostomy	3 (8.8)	6 (9.5)	>0.99
Outcome post-LT			
Biliary complications	8 (23.5)	6 (9.5)	0.06
Vascular complications	5 (14.7)	8 (12.7)	0.78
Early allograft dysfunction (EAD)	15 (44.1)	15 (23.8)	**0.04**
Prolonged postoperative steroids (>7 days)	3 (8.8)	9 (14.3)	0.44

LT, liver transplantation; BMI, body mass index; cACLD, compensated Advanced Chronic Liver Disease; N-AD, non-acute decompensation; AD, acute decompensation; ACLF, acute on chronic liver failure; ALF, acute liver failure; OF, organ failure; ICU, intensive care unit; MELD, Model for End-stage Liver Disease; SOFA, Sequential Organ Failure Assessment; DCD, donation after circulatory death; PRBC: packed red blood cells. Values are expressed as median [IQR] or n (%). Comparisons were performed using the Mann–Whitney U test or the Chi-square test, as appropriate. Bold values indicate statistical significance (p < 0.05).

**FIGURE 2:**
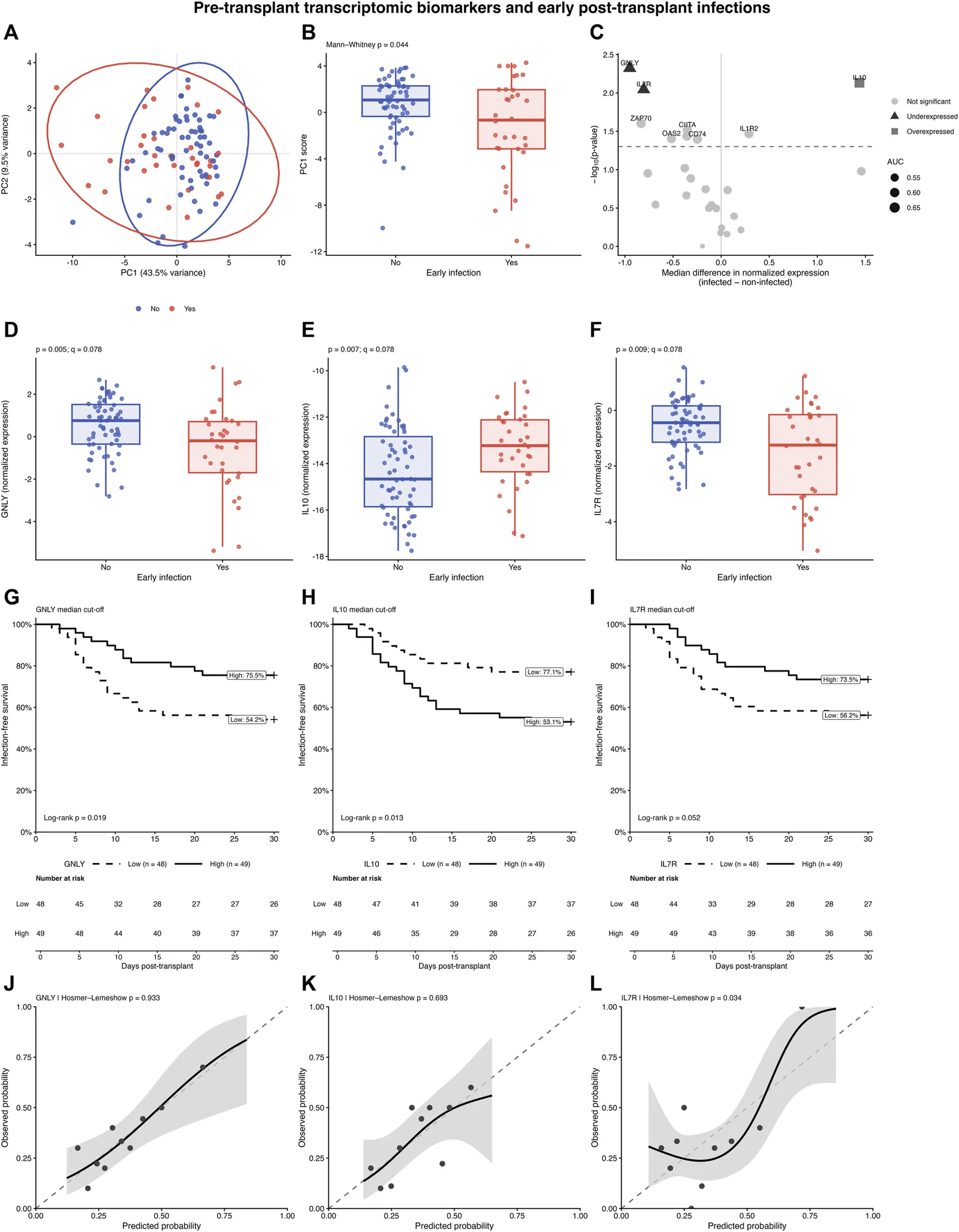
Pre-transplant transcriptomic biomarkers and early post-transplant infections. **(A)** PCA biplot colored according to early post-transplant infection status. Confidence ellipses are shown for each group. **(B)** PC1 score according to infection status (Wilcoxon rank-sum test p = 0.044). **(C)** Volcano plot representing the median difference in normalized expression between infected and non-infected patients (x-axis) and the −log10(p-value) (y-axis); point size represents the AUC. The dashed grey horizontal line indicates the nominal significance threshold (p < 0.05). Genes reaching adjusted significance (q < 0.10) are distinguished using distinct monochrome symbols: black triangles indicate genes underexpressed in infected patients, whereas dark grey squares indicate genes overexpressed in infected patients; non-significant genes are shown as light grey circles. **(D–F)** Normalized expression of GNLY, IL10 and IL7R according to infection status. Nominal p-values and Benjamini–Hochberg adjusted q-values are indicated. **(G–I)** Kaplan–Meier 30-day infection-free survival curves according to cohort median expression of GNLY, IL10 and IL7R, respectively. Patients were dichotomized using cohort median expression values. Low-expression groups are shown with dashed black lines and high-expression groups with solid black lines. Thirty-day infection-free survival rates are indicated for each group; log-rank p-values are shown. **(J–L)** Calibration plots for individual logistic regression models using GNLY, IL10 and IL7R, respectively. The solid black curve represents a generalized additive model (GAM) smoother, the grey shaded area represents its 95% confidence interval, filled grey points represent observed event rates across Hosmer–Lemeshow groups, and the dashed grey line represents perfect calibration. Hosmer–Lemeshow p-values are indicated on each panel. Gene expression values were normalized to reference genes. GAM, generalized additive model; LT, liver transplantation.

On the PCA biplot, samples from infected and non-infected patients showed overlapping distributions with partially separated confidence ellipses (Figure 2A). However, PC1 score was significantly lower in infected patients compared to non-infected patients (p = 0.044, uncorrected, Figure 2B), suggesting a possibly more pronounced pre-LT immune deficit in patients who subsequently developed infections (this comparison was not adjusted for multiple testing and should be interpreted as borderline).

Volcano plot analysis identified three genes reaching adjusted significance (q < 0.10 and AUC > 0.65): GNLY and IL7R were downregulated in infected patients, whereas IL10 was upregulated (Figure 2C). These genes were retained as candidate biomarkers for downstream analyses. For all 26 transcriptomic markers, univariate Wilcoxon rank-sum p-values, Benjamini–Hochberg adjusted q-values, and ROC-derived AUCs are reported in Supplementary Table 2.

Individual boxplots confirmed differential expression of IL10, GNLY and IL7R between infected and non-infected patients (Figures 2D–F). ROC analyses demonstrated comparable discriminative performances for IL10, GNLY and IL7R, with AUCs of 0.67 [0.55–0.78], 0.67 [0.56–0.79], and 0.66 [0.54–0.79], respectively. MELD and SOFA scores did not reach significance for predicting early infection (AUC 0.61 [0.49–0.74], p = 0.07 and 0.60 [0.47–0.72], p = 0.11).

Kaplan-Meier analyses using cohort medians as thresholds demonstrated significantly reduced 30-day infection-free survival in patients with low GNLY expression (54% vs. 76%, p = 0.019, Figure 2G) and with high IL10 expression (53% vs. 77%, p = 0.013, Figure 2H). IL7R dichotomized at the median showed a borderline association with infection-free survival (56% vs. 73%, p = 0.052, Figure 2I).

Calibration analyses of univariable logistic regression models demonstrated good agreement between predicted probabilities and observed infection rates for GNLY (Hosmer–Lemeshow, p = 0.93) and IL10 (p = 0.69). In contrast, IL7R showed borderline calibration (p = 0.03), with a non-linear pattern on the calibration plot suggesting heterogeneity across predicted probability ranges (Figures 2J–L).

### Association between immune markers and survival post-LT

Seven patients (7.2%) died within 1 year of LT; six deaths were infection-related and one resulted from refractory hemodynamic failure within 24 h post-LT. Regarding clinical characteristics, non-survivors tended to be younger, more frequently admitted to the ICU at the time of LT (57% vs. 18%), and had higher MELD (28 vs. 20) and SOFA scores (6 vs. 5); none of these differences reached statistical significance (Table 2). Among perioperative factors, intraoperative transfusion requirements were significantly higher in non-survivors (median 8 [5–9] vs. 2 [0–5] units, p < 0.001) and EAD was more frequent (71% vs. 28%, p = 0.03, Table 2).

**TABLE 2:** Recipient’s characteristics and severity score according to one-year survival post-LT.

Recipient’s characteristics	Non-survivors n = 7	Survivors n = 90	p
Age (years)	45 [44–56]	56 [48–62]	0.06
Sex (male)	4 (57.1)	74 (82.2)	0.26
Baseline severity scores			
Clinical presentation	​	​	0.12
cACLD	0 (0.0)	20 (22.2)	​
N-AD	0 (0.0)	23 (25.6)	​
AD	3 (42.9)	18 (20.0)	​
ACLF	3 (42.9)	26 (28.9)	​
ALF	1 (14.2)	3 (3.3)	​
OF (ACLF/ALF)	4 (57.1)	29 (32.2)	0.35
Number of organ failures	2 [0–4]	0 [0–2]	0.14
MELD score	28 [20–40]	20 [13–27]	0.08
SOFA score	6 [4–14]	5 [2–6]	0.11
Hospitalisation status at LT			
ICU	4 (57.1)	16 (17.8)	0.08
Hepatology ward	0 (0.0)	12 (13.3)	​
Outpatient	3 (42.9)	62 (68.9)	​
Donors characteristics			
Age (years)	67 [49–71]	66 [51–73]	0.81
Sex (male)	2 (28.6)	60 (66.7)	0.09
BMI (kg/m^2^)	28 [24–35]	25 [22–28]	0.14
DCD	0 (0.0)	17 (18.9)	0.35
Causes of donor death	​	​	0.20
Cerebrovascular	4 (57.1)	44 (48.9)	​
Traumatic	1 (14.3)	16 (17.8)	​
Anoxic	1 (14.3)	29 (32.2)	​
Meningitis	1 (14.3)	1 (1.1)	​
Surgery characteristics			
PRBC transfusion	8 [5–9]	2 [0–5]	**<0.001**
Warm ischemia	40 [32–53]	37 [26–41]	0.29
Cold ischemia	400 [344–491]	394 [325–469]	0.75
Hepaticojejunostomy	1 (14.3)	8 (8.9)	​
Outcome post-LT			
Biliary complications	1 (14.3)	13 (14.4)	>0.99
Vascular complications	2 (28.6)	11 (12.2)	0.24
Early allograft dysfunction (EAD)	5 (71.4)	25 (27.8)	0.03
Prolonged postoperative steroids (>7 days)	0 (0.0)	12 (13.3)	0.59

LT, liver transplantation; cACLD, compensated Advanced Chronic Liver Disease; N-AD, non-acute decompensation; AD, acute decompensation; ACLF, acute on-chronic liver failure; ALF, acute liver failure; ICU, intensive care unit; OF, organ failure; MELD, Model for End-stage Liver Disease; SOFA, Sequential Organ Failure Assessment; DCD, donation after circulatory death; PRBC, packed red blood cells. Values are expressed as median [IQR] or n (%). Comparisons were performed using the Mann–Whitney U test or the Chi-square test, as appropriate. Bold values indicate statistical significance (p < 0.05).

**FIGURE 3:**
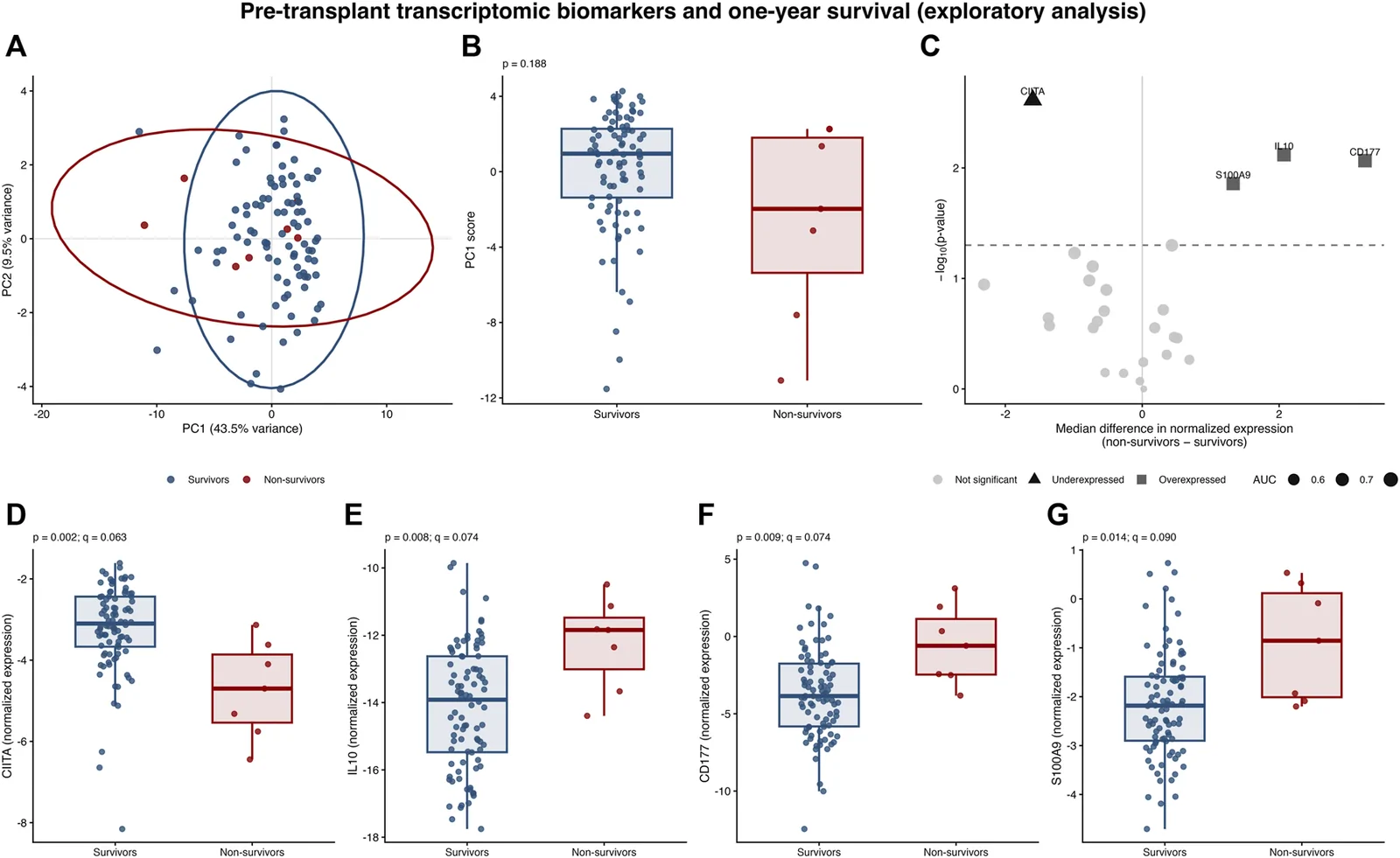
Pre-transplant transcriptomic biomarkers and one-year survival (exploratory analysis). **(A)** PCA biplot colored according to one-year survival status. Confidence ellipses are shown for each group. **(B)** PC1 score according to one-year survival status (Wilcoxon rank-sum test p = 0.188). **(C)** Volcano plot representing the median difference in normalized expression between non-survivors and survivors (x-axis) and the −log10(p-value) (y-axis); dot size represents the AUC. The dashed grey horizontal line indicates the nominal significance threshold (p < 0.05). Genes reaching adjusted significance (q < 0.10) are distinguished using distinct monochrome symbols: black triangles indicate genes underexpressed in non-survivors, whereas dark grey squares indicate genes overexpressed in non-survivors; non-significant genes are shown as light grey circles. **(D–G)** Normalized expression of CIITA, IL10, CD177 and S100A9 according to one-year survival status. Nominal p-values and Benjamini–Hochberg adjusted q-values are indicated. Given the limited number of events (n = 7 deaths), all analyses should be interpreted with caution. Gene expression values were normalized to reference genes. Given the limited number of deaths (n = 7), Kaplan-Meier analyses are provided in Supplementary Figure 4. LT, liver transplantation.

On the PCA biplot, samples from survivors and non-survivors showed largely overlapping distributions (Figure 3A), and PC1 score did not differ significantly between groups (p = 0.188, Figure 3B). This binary comparison discards timing and censoring information and should therefore be interpreted with caution; it suggests, but does not establish, that the global immune signature captured by PC1 is less discriminant for one-year mortality than for early infections. Volcano plot analysis identified four genes meeting the predefined exploratory selection criteria (q < 0.10 and AUC > 0.65): CIITA was underexpressed in non-survivors, whereas IL10, CD177 and S100A9 were overexpressed (Figure 3C). AUC values, nominal p-values and Benjamini–Hochberg adjusted q-values for all 26 transcriptomic markers are reported in Supplementary Table 3. These four genes were retained as candidate biomarkers for further exploratory analyses. Individual boxplots confirmed differential expression of CIITA, IL10, CD177 and S100A9 between survivors and non-survivors (Figures 3D–G). ROC curve analyses showed AUCs ranging from 0.79 to 0.85 for these candidate markers (Supplementary Table 3).

Given the limited number of deaths (n = 7), survival analyses were considered exploratory. Kaplan-Meier analyses using cohort median thresholds showed significantly lower one-year overall survival among patients with low CIITA expression (87.5% vs. 98.0%, p = 0.049), while similar trends were observed for high IL10, CD177 and S100A9 expression (Supplementary Figure 4).

## Discussion

In this exploratory prospective study of 97 LT recipients, pre-transplant whole blood transcriptomic profiling of 26 immune-related genes appears to capture a progressive gradient of immune dysfunction across liver disease severity stages, consistent with the known features of CAID. Beyond this characterization, specific transcriptomic markers were associated with post-LT outcomes, early infections and, in a preliminary exploratory analysis, one-year survival. This suggests that immune profiling may capture prognostic information beyond disease severity alone, although whether it provides added value over clinical scores available prior to LT, which was not assessed here, is an interesting avenue for further research.

The present findings suggest that pre-transplant whole blood transcriptomic profiling of a 26 immune-related gene signature may represent a promising approach for characterizing CAID in patients with end-stage liver disease. In this study, the dominant axis of transcriptomic variability decreased progressively from cACLD to ALF, capturing the CAID severity continuum in an unsupervised manner. Loading analysis identified two opposing gene clusters: genes involved in monocyte antigen presentation and immune effector functions, including CIITA, CX3CR1, CD74, TAP2, ZAP70, IL7R and GNLY were progressively downregulated, while markers of innate myeloid activation and anti-inflammatory signaling, CD177, S100A9 and IL10, were upregulated with increasing severity. This bidirectional pattern is consistent with the established features of CAID, characterized by simultaneous adaptive immune exhaustion and innate inflammatory dysregulation [1, 8, 9]. The magnitude of transcriptomic changes was comparable to that observed in this study for mHLA-DR across severity stages or in other immune alterations described previously [8, 19, 20], supporting the biological validity of the panel.

It is well established that patients with ACLF and ALF experience the highest rates of adverse post-transplant outcomes, including infections and mortality [4, 21]. However, conventional severity scores, such as the MELD and SOFA scores, primarily reflect pre-transplant disease severity in the recipient and have limited ability to predict post-LT infectious complications or guide individualized perioperative management. While underlying immunological dysfunctions (CAID) are well documented in cirrhotic patients [1, 8, 9], their association with post-transplant complications remains poorly investigated. Several studies have reported associations between pre-transplant immune status and post-LT outcomes using protein-level or cellular immune markers [5–7]. None of these biomarkers have been validated for use in clinical practice to guide personalized post-LT management strategies. In a previous report from our group, post-LT mHLA-DR trajectories were independently associated with early infectious complications and one-year survival [11], suggesting that dynamic immune monitoring may hold prognostic relevance in this setting. Yet, conventional pre-LT immune markers, including mHLA-DR expression and lymphocyte counts, failed to predict post-LT outcomes in this previous study, underscoring the limitations of standard immunological parameters and the need for more comprehensive pre-transplant immune characterization. In this regard, whole blood transcriptomic profiling could be more effective than clinical scores at stratifying patients according to their prognosis in sepsis [22, 23], although its applicability in the context of LT remains unexplored.

According to Principal Component Analysis, the dominant axis of pre-LT transcriptomic variability showed only limited discrimination between patients who did and did not develop post-LT infections. Volcano plot analysis revealed that three main pre-LT transcriptomic markers (IL7R, IL-10 and GNLY) were dysregulated in patients who developed post-LT infections. Survival curves according to the median thresholds for each of these genes suggest that their pre-LT expression may identify patients at increased risk of early post-LT infections. Calibration analyses showed good agreement between predicted and observed infection probabilities for GNLY and IL10, whereas calibration was suboptimal for IL7R, suggesting that the predictive performance of individual genes within the signature is heterogeneous, and that the model’s accuracy may rely more on the combined expression pattern of several genes than on the contribution of any single gene. In addition, the mRNA levels of three additional genes (CIITA, CD177 and S100A9) as well as IL-10 mRNA levels may be associated with one-year mortality following liver transplantation. These mRNA alterations closely mirrored those observed in sepsis-induced immunosuppression. CIITA, whose mRNA levels are reduced, encodes proteins involved in MHC class II expression, aligning with the well-described decrease in mHLA-DR expression in sepsis [24, 25]. Increased IL10 mRNA, a key immunosuppressive cytokine, has also been reported in sepsis along with elevated circulating IL-10 levels [26, 27]. Similarly, decreased IL7R mRNA expression reflects the findings in septic patients and is consistent with the profound lymphopenia observed in this population [28, 29], as IL-7 plays a critical role in promoting T-cell proliferation. Elevated CD177 is considered a marker of neutrophil immaturity, another hallmark of sepsis-induced immunosuppression [12, 30], whereas decreased GNLY expression reflects impaired cytotoxic functionality [31]. These findings, which closely align with the mechanisms of sepsis-induced immunosuppression, suggest that maladaptive immune reprogramming driven by progressive cirrhosis may persist even after LT. From a pathophysiological standpoint, further investigation is warranted to explore the potential causal link between pre-transplant immunosuppression and unfavorable post-transplant outcomes. From a clinical perspective, pre-LT immunological monitoring could facilitate the identification of patients who are at high risk of post-LT infection and pave the way for individualized post-transplant monitoring strategies. These preliminary findings require further validation through the recruitment of a larger cohort.

This study had several limitations. This is an exploratory study with a single-center design and modest sample size. This may have limited the generalizability of our results. In particular, the modest sample size and the limited number of outcome events precluded multivariable analyses adjusting marker–outcome associations for pre-transplant disease severity or other clinical confounders; consequently, this exploratory study cannot determine whether the identified transcriptomic markers add prognostic information beyond established clinical severity scores. Because candidate genes were selected in part on the basis of their AUC (q < 0.10 and AUC > 0.65), and the same AUCs are reported here as a measure of their performance, these estimates are likely optimistic and should be expected to be lower when evaluated in an independent validation cohort.

Thus, validation cohorts would be valuable to confirm the predictive value of these markers with stratification according to pre-LT disease severity (including ACLF grades), liver disease etiology and immunosuppressive regimens. In order to ascertain the additional value of pre-LT transcriptomic signatures, comparisons between validated post-LT prognosis scores - integrating donor and LT data - will also be necessary. Further studies on Acute Liver Failure may also be needed to confirm these results in this subgroup without underlying liver disease. Given the low number of patients and their similar clustering in PCA, we decided to analyze ACLF and ALF patients together, as they are both characterized by organ failure and similar features of innate immune dysfunction [32, 33]. Although the targeted transcriptomic panel used in this study is based on well-established immunological knowledge and is designed to provide a global overview of the immune response, encompassing genes involved in various stages of immune activation and regulation, it may not capture broader immune alterations. More comprehensive approaches, such as RNA sequencing, could offer additional insights and uncover novel pathways involved in post-transplant outcomes. The variable interval between the last pre-LT transcriptomic sample and transplantation (median 9 days [1–36]) reflects the constraints of a biobanking protocol designed for repeat immune monitoring across disease stages. In biologically dynamic populations such as ACLF, the immune status may evolve substantially over days. Thus, the interval between sampling day and LT date may result in a moderate distortion of the results. Sensitivity analyses restricted to samples collected within a shorter interval were not feasible given the sample size, and this temporal variability should be considered when interpreting our findings.

Overall, our results suggest that novel mRNA biomarkers associated with cirrhosis-associated immune dysfunction (CAID) may be valuable pre-LT tools to predict unfavorable outcomes following LT. Further studies will be necessary to confirm these preliminary results and explore the additional value of this immune assessment in clinical practice. Beyond the significance of these findings, which suggest that pre-transplant immune alterations may be linked to post-transplant clinical outcomes and warrant further investigation, this study also has important clinical implications. From a pragmatic perspective, integrating transcriptomic profiling into the pre-transplant evaluation process could markedly improve patient stratification and perioperative management. Specifically, identifying patients at high risk for adverse outcomes may enable more personalized monitoring, targeted immunomodulatory strategies, and better-informed decisions regarding the timing and suitability of transplantation.

## Data Availability

The raw data supporting the conclusions of this article will be made available by the authors, without undue reservation.

## Glossary

ACLF: acute-on-chronic liver failure
ACLD: advanced chronic liver disease
AD: acute decompensation
ALD: alcohol-related liver disease
ALF: acute liver failure
AUC: area under the curve
CAID: cirrhosis-associated immune dysfunction
CIITA: class II major histocompatibility complex transactivator
CLIF: chronic liver failure
cACLD: compensated advanced chronic liver disease
CD177: cluster of differentiation 177
CD74: cluster of differentiation 74
CX3CR1: C-X3-C motif chemokine receptor 1
DCD: donation after circulatory death
GAM: generalized additive model
GNLY: granulysin
HLA-DR: human leukocyte antigen–DR isotype
ICU: intensive care unit
IL7R: interleukin 7 receptor
IL10: interleukin 10
IPP: immune profiling panel
IQR: interquartile range
LT: liver transplantation
MASH: metabolic dysfunction-associated steatohepatitis
MELD: model for end-stage liver disease
mHLA-DR: monocytic human leukocyte antigen–DR isotype
N-AD: non-acute decompensation
OF: organ failure
OR: odds ratio
PCA: principal component analysis
qPCR: quantitative polymerase chain reaction
ROC: receiver operating characteristic
RT-qPCR: reverse transcription quantitative polymerase chain reaction
SOFA: sequential organ failure assessment
TAP2: transporter associated with antigen processing 2
TNF: tumor necrosis factor
ZAP70: zeta-chain associated protein kinase 70
