# Supplementary material for: Pre-transplant whole blood transcriptomic profiling identifies mRNAs linked to cirrhosis-associated immune dysfunction as candidate predictors of clinical outcomes after liver transplantation: an exploratory study
Source: Transpl Int. 2026 Aug 28;39:16292. doi: 10.3389/ti.2026.16292 (PMC13563205; doi:10.3389/ti.2026.16292)
Supplement: Supplementary file 1 [file Supplementaryfile1.docx]

**SUPPLEMENTARY FIGURE 1**

**The immune profiling panel.** The figure illustrates the selected markers of the panel that includes 26 target genes and describes the different pathways targeted. The panel of markers was selected to target different aspects of the immune responses (innate and adaptive), several immune functions (pro- and anti-inflammatory cytokines) and immune pathways.

OAS2:2'-5'-Oligoadénylate synthétase 2; TNF: Tumor Necrosis Factor; IFN-γ: Interféron gamma; ADGRE3: Adhesion G Protein-Coupled Receptor E3; IP10: Interferon gamma-induced protein 10; CD177: cluster of differentiation 177 molecule; C3AR1: Complement Component 3a Receptor 1; GNLY: Granulysin; MDC1: Mediator of DNA Damage Checkpoint Protein 1; S100A9: S100 calcium-binding protein A9 (calprotectine); IL1R2: Interleukin 1 Receptor Type 2; IL1RN: Interleukin 1 Receptor Antagonist; IL10: Interleukin 10; CX3CR1: CX3C chemokine receptor 1; CIITA: Class II Major Histocompatibility Complex Transactivator; TAP2: Transporter associated with Antigen Processing 2; CD74: cluster of differentiation 74; CD3D: CD3 delta chain; ZAP70: Zeta-chain Associated Protein Kinase 70; IL7R: Interleukin 7 Receptor; TDRD9: Tudor Domain Containing 9; ARL14EP: ARL14 Effector Protein; CD274: Cluster of differentiation 274 molecule (PD-L1); CTLA4: Cytotoxic T-Lymphocyte Antigen 4; BPGM: Bisphosphoglycerate Mutase; CCNB1IP1: Cyclin B1 Interacting Protein 1

**SUPPLEMENTARY FIGURE 2**

**Principal component analysis of the 26-gene immune transcriptomic panel before liver transplantation.**

PCA was performed using normalized whole-blood expression levels of the 26-gene immune panel. Each grey dot represents one patient. Arrows indicate the top 10 genes contributing to PC1 and the top 3 genes contributing to PC2, based on variable–component correlations. Genes are color-coded according to their predominant contribution to PC1 (orange) or PC2 (blue). PC1 explained 43.5% of total variance and PC2 explained 9.5%.

**SUPPLEMENTARY FIGURE 3**

**Significant additional pre-transplant immune markers according to liver disease severity.**
Panels A–Q display normalized expression levels of transcriptomic markers identified as significantly associated with liver disease severity after Benjamini–Hochberg correction for multiple testing (FDR q<0.10). Patients were grouped according to liver disease severity categories: compensated advanced chronic liver disease (cACLD), non-acute decompensation (NAD), acute decompensation (AD), acute-on-chronic liver failure (ACLF), and acute liver failure (ALF). Panel R shows pre-transplant monocytic HLA-DR expression (mHLA-DR, AB/C), and panel S shows absolute lymphocyte counts. Global comparisons between severity groups were performed using Kruskal–Wallis tests, with nominal p-values and adjusted q-values indicated on each panel. Gene expression values were normalized to reference genes. FDR, false discovery rate.

**SUPPLEMENTARY FIGURE 4**

**One-year overall survival according to pre-transplant transcriptomic marker expression.**
Kaplan–Meier survival curves showing 1-year overall survival after liver transplantation according to pre-transplant expression levels of CIITA (A), IL10 (B), CD177 (C) and S100A9 (D). Patients were dichotomized according to the median expression level of each marker. Low-expression groups are shown with dashed black lines and high-expression groups with solid black lines. P-values were calculated using log-rank tests.
