# Supplementary material for: Pre-transplant whole blood transcriptomic profiling identifies mRNAs linked to cirrhosis-associated immune dysfunction as candidate predictors of clinical outcomes after liver transplantation: an exploratory study
Source: Transpl Int. 2026 Aug 28;39:16292. doi: 10.3389/ti.2026.16292 (PMC13563205; doi:10.3389/ti.2026.16292)
Supplement: Supplementary file 2 [file Supplementaryfile2.docx]

**Supplementary Materials**

**Methods**

**Diagnostic criteria for infections according to the American Society of Transplantation Recommendations for Screening, Monitoring and Reporting of Infectious Complications in Immunosuppression Trials in Recipients of Organ Transplantation**

Pyelonephritis was defined by the presence of a positive urine culture and pyuria in association with tenderness at costovertebral angle with fever and/or positive blood culture.

Bacterial pneumonia was defined by the presence of one or more of the following symptoms: cough, hemoptysis, fever, dyspnea (or increased oxygen requirement), pleuritic chest pain plus chest X-ray or CT chest findings of new pulmonary parenchymal disease. A definite diagnosis was based upon meeting the above criteria in combination with the presence of a positive sputum or BAL culture. In the absence of one or more of these criteria, the patient should be considered to have an episode of probable pneumonia.

Intra-abdominal infection could be diagnosed based on results of CT or ultrasound of the abdomen as well as by identification at laparotomy. Microbiologic evidence included the presence of a positive culture obtained in sterile fashion (i.e. needle biopsy, operative specimen or a new drain).

Bacteremia must be documented by a positive blood culture and should be characterized by its source or accompanying syndrome. Examples of potential sources and syndromes include central venous catheter-related bacteremia, pneumonia, intra-abdominal infection, skin/soft tissue infection or meningitis. Contamination should be differentiated from true bacteremia by clinical presentation and if possible by repeated peripheral blood culture rather than from previously placed indwelling catheters. A single positive culture for coagulase negative staphylococcus in the absence of any symptoms is often a contaminant and should not be classified as a definitive bacteremia.

**Early allograft dysfunction**

Early allograft dysfunction (EAD) was defined according to Olthoff et al. as the presence of at least one of the following criteria within the first 7 postoperative days: alanine aminotransferase (ALT) or aspartate aminotransferase (AST) >2,000 IU/L, bilirubin ≥10 mg/dL on postoperative day 7, or international normalized ratio (INR) ≥1.6 on postoperative day 7 [18].
