# Supplementary material for: Pre-transplant whole blood transcriptomic profiling identifies mRNAs linked to cirrhosis-associated immune dysfunction as candidate predictors of clinical outcomes after liver transplantation: an exploratory study
Source: Transpl Int. 2026 Aug 28;39:16292. doi: 10.3389/ti.2026.16292 (PMC13563205; doi:10.3389/ti.2026.16292)
Supplement: Supplementary file 3 [file Supplementaryfile3.docx]

**Supplementary Materials**

**Supplementary results**

**Liver disease etiology:**

Among the 11 patients with viral etiologies, six had hepatitis B virus (HBV)-related cirrhosis: four had undetectable viral loads, one patient with acute decompensation had a detectable but non-quantifiable HBV viral load, and one patient with ACLF had a quantifiable positive viral load at the time of assessment. The remaining five patients had hepatitis C virus (HCV)-related cirrhosis, all with undetectable viral loads following antiviral treatment.

**Post-LT infections:**

Enterobacter cloacae (n = 1) and Staphylococcus epidermidis were responsible for the two reported cases of bacteremia. When microbial identification was available for patients with intra-abdominal infections (n = 19), the pathogens identified were Enterococcus spp (n = 7), Klebsiella pneumoniae (n = 1), Bacillus cereus (n = 1), Escherichia coli (n = 1), Staphylococcus spp (n = 4) and Cutibacterium acnes (n = 1). Pathogens identified in pneumonia were Klebsiella pneumoniae (n = 1), Pseudomonas aeruginosa (n = 1), Staphylococcus spp (n = 4), Stenotrophomonas maltophilia (n = 1), Enterococcus spp (n = 2) and Burkholderia spp (n = 1). The fungal infections were as follows: two cases of candidemia and one case of Candida peritonitis. Both viral infections were cytomegalovirus reactivations.
