# Supplementary material for: Pre-transplant whole blood transcriptomic profiling identifies mRNAs linked to cirrhosis-associated immune dysfunction as candidate predictors of clinical outcomes after liver transplantation: an exploratory study
Source: Transpl Int. 2026 Aug 28;39:16292. doi: 10.3389/ti.2026.16292 (PMC13563205; doi:10.3389/ti.2026.16292)
Supplement: Supplementary file 4 [file Table1.docx]

**Supplementary Table 1**. **Expression of the 26-gene immune transcriptomic panel according to cirrhosis etiology.**

|  | **pre-LT Immune Panel** | **Auto-immune (n=8)** | **ALD (n=68)** | **Viral (n=11)** | **p-value^1^** | **q-value^2^** | | |
| --- | --- | --- | --- | --- | --- | --- | --- | --- |
|  | ADGRE3 | 3.44 [2.86–3.80] | 2.93 [2.62–3.35] | 2.80 [2.50–3.00] | 0.076 | 0.329 | |  |
|  | ARL14EP | -7.49 [-8.21–-7.28] | -7.52 [-7.86–-7.12] | -7.57 [-8.31–-6.73] | 0.876 | 0.913 | |  |
|  | BPGM | 6.61 [6.09–7.34] | 6.14 [5.57–7.26] | 6.13 [4.98–6.40] | 0.580 | 0.718 | |  |
|  | C3AR1 | -0.58 [-1.27–-0.13] | -0.58 [-1.28–-0.16] | -0.73 [-1.83–-0.63] | 0.246 | 0.437 | |  |
|  | CCNB1IP1 | -2.40 [-2.74–-2.33] | -2.18 [-2.54–-1.89] | -1.92 [-2.72–-1.77] | 0.153 | 0.414 | |  |
|  | CD177 | -2.67 [-3.81–-0.66] | -3.65 [-5.74–-1.68] | -4.88 [-6.62–-4.29] | **0.047** | 0.327 | |  |
|  | CD274 | -0.69 [-1.05–-0.31] | -0.93 [-1.94–-0.54] | -1.47 [-1.70–-0.82] | 0.215 | 0.430 | |  |
|  | CD3D | -0.95 [-1.26–0.09] | -0.06 [-0.84–0.23] | 0.14 [0.00–0.36] | 0.149 | 0.414 | |  |
|  | CD74 | 3.51 [3.13–3.60] | 3.47 [2.63–3.95] | 3.91 [3.66–4.23] | **0.048** | 0.327 | |  |
|  | CIITA | -3.36 [-3.59–-2.57] | -3.18 [-3.87–-2.66] | -2.32 [-3.13–-2.06] | 0.050 | 0.327 | |  |
|  | CTLA4 | -1.37 [-1.77–-0.38] | -0.87 [-1.53–-0.41] | -0.62 [-1.10–-0.29] | 0.361 | 0.489 | |  |
|  | CX3CR1 | -2.08 [-2.44–-1.62] | -1.89 [-2.31–-1.52] | -1.79 [-2.67–-1.38] | 0.878 | 0.913 | |  |
|  | GNLY | -0.27 [-0.83–1.13] | 0.36 [-0.72–1.34] | 0.93 [-0.82–1.49] | 0.642 | 0.759 | |  |
|  | IFNɣ | -4.32 [-4.92–-3.28] | -3.27 [-4.05–-2.59] | -2.53 [-4.89–-1.95] | 0.178 | 0.414 | |  |
|  | IL10 | -12.82 [-14.88–-12.38] | -13.77 [-15.21–-12.52] | -15.78 [-16.99–-12.50] | 0.191 | 0.414 | |  |
|  | IL1R2 | 3.21 [2.51–3.65] | 2.94 [2.34–3.46] | 2.37 [1.98–3.24] | 0.376 | 0.489 | |  |
|  | IL1RN | -4.19 [-4.91–-3.75] | -4.77 [-5.33–-4.13] | -5.07 [-5.96–-4.94] | **0.039** | 0.327 | |  |
|  | IL7R | -0.60 [-1.79–-0.15] | -0.53 [-1.68–0.25] | -0.26 [-1.17–0.01] | 0.856 | 0.913 | |  |
|  | IP10 | -5.23 [-5.73–-3.17] | -6.33 [-7.83–-5.07] | -6.13 [-7.19–-3.64] | 0.133 | 0.414 | |  |
|  | MDC1 | -1.33 [-1.49–-1.09] | -1.02 [-1.34–-0.54] | -1.02 [-1.18–-0.59] | 0.252 | 0.437 | |  |
|  | OAS2 | -3.82 [-4.63–-3.00] | -4.41 [-4.92–-3.72] | -4.13 [-4.42–-3.21] | 0.167 | 0.414 | |  |
|  | S100A9 | -1.79 [-2.18–-1.70] | -2.19 [-2.89–-1.58] | -2.50 [-3.44–-1.59] | 0.296 | 0.452 | |  |
|  | TAP2 | -0.25 [-0.50–-0.04] | -0.47 [-0.97–-0.06] | -0.10 [-0.57–0.08] | 0.286 | 0.452 | |  |
|  | TDRD9 | -1.78 [-2.18–-1.56] | -2.19 [-3.03–-1.28] | -2.92 [-3.67–-1.99] | 0.063 | 0.327 | |  |
|  | TNF | -0.26 [-1.26–0.05] | -0.39 [-0.73–0.00] | -0.40 [-0.87–0.04] | 0.929 | 0.929 | |  |
|  | ZAP70 | 1.26 [0.42–1.74] | 1.70 [0.85–2.20] | 2.05 [0.84–2.43] | 0.348 | 0.489 | |  |
| Expression levels of the 26 immune-related genes were compared across the main cirrhosis etiologies before liver transplantation. Patients with acute liver failure (ALF), metabolic-associated steatohepatitis (MASH), and other rare etiologies were excluded from this analysis because of limited sample sizes. Data are presented as median [interquartile range]. Statistically significant p-values and q-values are shown in bold. ¹Kruskal-Wallis test (significant if p<0.05). ²False discovery rate correction for multiple testing (significant if q<0.10). | | | | | | |  |  |
|  | | | | | | |  |  |
