# Supplementary material for: Pre-transplant whole blood transcriptomic profiling identifies mRNAs linked to cirrhosis-associated immune dysfunction as candidate predictors of clinical outcomes after liver transplantation: an exploratory study
Source: Transpl Int. 2026 Aug 28;39:16292. doi: 10.3389/ti.2026.16292 (PMC13563205; doi:10.3389/ti.2026.16292)
Supplement: Supplementary file 5 [file Table2.docx]

**Supplementary Table 2**. **Association between pre-transplant expression levels of the 26-gene immune transcriptomic panel and post-transplant infections.**

|  | **pre-LT Immune Panel** | **Post-LT infections**  **n=34** | **No post-LT infections**  **n=63** | **p-value^1^** | **q-value^2^** | **AUC** |  |  |
| --- | --- | --- | --- | --- | --- | --- | --- | --- |
|  | ADGRE3 | 2.82 (2.35, 3.29) | 2.94 (2.64, 3.38) | 0.3 | 0.4 | 0.56 [0.44-0.67] | |  |
|  | ARL14EP | -7.77 (-7.93, -7.39) | -7.39 (-7.88, -7.11) | 0.10 | 0.2 | 0.60 [0.49-0.72] | |  |
|  | BPGM | 6.08 (4.96, 7.07) | 6.40 (5.77, 7.19) | 0.13 | 0.2 | 0.59 [0.47-0.72] | |  |
|  | C3AR1 | -0.64 (-1.17, -0.23) | -0.70 (-1.33, -0.25) | 0.7 | 0.7 | 0.52 [0.40-0.65] | |  |
|  | CCNB1IP1 | -2.30 (-2.90, -1.88) | -2.20 (-2.41, -1.92) | 0.3 | 0.4 | 0.57 [0.43-0.69] | |  |
|  | CD177 | -2.49 (-5.92, -0.29) | -3.95 (-5.42, -2.33) | 0.10 | 0.2 | 0.60 [0.47-0.72] | |  |
|  | CD274 | -1.13 (-1.78, -0.52) | -0.93 (-1.71, -0.56) | 0.3 | 0.4 | 0.50 [0.38-0.63] | |  |
|  | CD3D | -0.19 (-1.43, 0.23) | -0.03 (-0.59, 0.24) | 0.10 | 0.2 | 0.58 [0.44-0.71] | |  |
|  | CD74 | 3.35 (2.06, 3.82) | 3.59 (3.06, 4.04) | 0.13 | 0.2 | 0.63 [0.51-0.74] | |  |
|  | CIITA | -3.38 (-4.12, -2.78) | -3.03 (-3.59, -2.43) | **0.030** | 0.13 | 0.63 [0.52-0.75] | |  |
|  | CTLA4 | -1.17 (-1.62, -0.40) | -0.81 (-1.41, -0.43) | 0.2 | 0.3 | 0.58 [0.45-0.70] | |  |
|  | CX3CR1 | -1.89 (-3.26, -1.46) | -1.90 (-2.32, -1.55) | 0.6 | 0.7 | 0.54 [0.40-0.67] | |  |
|  | GNLY | -0.19 (-1.70, 0.71) | 0.76 (-0.35, 1.52) | **0.005** | **0.08** | **0.67 [0.55-0.78]** | |  |
|  | IFNɣ | -3.91 (-5.40, -2.31) | -3.22 (-4.11, -2.60) | 0.3 | 0.4 | 0.57 [0.43-0.70] | |  |
|  | IL10 | -13.23 (-14.35, -12.12) | -14.67 (-15.86, -12.84) | **0.007** | **0.08** | **0.67 [0.56-0.77]** | |  |
|  | IL1R2 | 3.17 (2.60, 4.37) | 2.88 (2.19, 3.41) | **0.034** | 0.13 | **0.63 [0.51-0.75]** | |  |
|  | IL1RN | -4.78 (-5.31, -4.01) | -4.91 (-5.37, -4.24) | 0.4 | 0.5 | 0.55 [0.42-0.68] | |  |
|  | IL7R | -1.25 (-3.03, -0.16) | -0.44 (-1.14, 0.16) | **0.009** | **0.08** | **0.66 [0.53-0.78]** | |  |
|  | IP10 | -6.74 (-8.65, -5.43) | -5.97 (-7.55, -4.91) | 0.11 | 0.2 | 0.60 [0.48-0.72] | |  |
|  | MDC1 | -1.08 (-1.79, -0.55) | -1.03 (-1.31, -0.65) | 0.3 | 0.4 | 0.56 [0.42-0.68] | |  |
|  | OAS2 | -4.62 (-5.08, -4.23) | -4.10 (-4.74, -3.45) | **0.039** | 0.13 | **0.63 [0.51-0.75]** | |  |
|  | S100A9 | -2.11 (-2.87, -0.44) | -2.18 (-2.84, -1.66) | 0.2 | 0.3 | 0.58 [0.45-0.71] | |  |
|  | TAP2 | -0.71 (-1.24, -0.18) | -0.36 (-0.69, 0.04) | **0.037** | 0.13 | **0.63 [0.51-0.75]** | |  |
|  | TDRD9 | -1.93 (-3.04, -0.94) | -2.13 (-2.89, -1.57) | 0.6 | 0.7 | 0.53 [0.41-0.65] | |  |
|  | TNF | -0.41 (-0.88, -0.04) | -0.40 (-0.74, -0.10) | 0.7 | 0.7 | 0.53 [0.40-0.65] | |  |
|  | ZAP70 | 0.89 (-0.21, 2.08) | 1.72 (1.06, 2.24) | **0.025** | 0.13 | **0.64 [0.51-0.76]** | |  |
| For each transcriptomic marker, expression levels measured before liver transplantation are presented according to the occurrence of post-transplant infection as median [interquartile range] in infected and non-infected patients. Group comparisons were performed using Wilcoxon rank-sum tests. False discovery rate (FDR) correction was applied for multiple testing. Discriminatory performance for infection prediction was assessed using receiver operating characteristic analyses and is reported as area under the curve (AUC) with 95% confidence intervals. Statistically significant p-values and q-values are shown in bold. AUC values are shown in bold when the 95% confidence interval does not include 0.50. ¹Wilcoxon rank-sum test (significant if p<0.05). ²False discovery rate correction for multiple testing (significant if q<0.10). ^3^Area Under the Curve. | | | | | | | | |
