# Supplementary material for: Pre-transplant whole blood transcriptomic profiling identifies mRNAs linked to cirrhosis-associated immune dysfunction as candidate predictors of clinical outcomes after liver transplantation: an exploratory study
Source: Transpl Int. 2026 Aug 28;39:16292. doi: 10.3389/ti.2026.16292 (PMC13563205; doi:10.3389/ti.2026.16292)
Supplement: Supplementary file 6 [file Table3.docx]

**Supplementary Table 3**. **Association between pre-transplant expression levels of the 26-gene immune transcriptomic panel and 1-year mortality after liver transplantation.**

| **pre-LT Immune Panel** | **Non-Survivors**  **n=7** | **Survivors**  **n=90** | **p-value^1^** | **q-value^2^** | **AUC^3^** |  |  |
| --- | --- | --- | --- | --- | --- | --- | --- |
| ADGRE3 | 1.94 (1.88, 2.79) | 2.93 (2.63, 3.39) | 0.059 | 0.2 | 0.72 [0.43-0.93] |  |  |
| ARL14EP | -7.60 (-7.81, -7.28) | -7.62 (-7.93, -7.13) | >0.9 | >0.9 | 0.50 [0.29–0.71] |  |  |
| BPGM | 5.71 (4.22, 8.84) | 6.25 (5.68, 7.13) | 0.7 | 0.8 | 0.54 [0.20–0.89] |  |  |
| C3AR1 | -1.33 (-1.83, -0.47) | -0.68 (-1.23, -0.23) | 0.2 | 0.4 | 0.63 [0.33-0.89] |  |  |
| CCNB1IP1 | -2.20 (-2.98, -1.91) | -2.22 (-2.54, -1.90) | 0.6 | 0.7 | 0.57 [0.27–0.85] |  |  |
| CD177 | -0.60 (-2.47, 1.13) | -3.85 (-5.82, -1.76) | **0.009** | **0.074** | **0.80 [0.66-0.93]** |  |  |
| CD274 | -0.65 (-1.20, -0.31) | -0.96 (-1.78, -0.55) | 0.2 | 0.4 | 0.65 [0.42-0.86] |  |  |
| CD3D | 0.43 (-0.93, 0.69) | -0.08 (-0.87, 0.22) | 0.3 | 0.5 | 0.61 [0.27–0.92] |  |  |
| CD74 | 2.85 (1.47, 3.49) | 3.57 (2.91, 3.97) | 0.078 | 0.2 | 0.70 [0.43-0.93] |  |  |
| CIITA | -4.70 (-5.54, -3.86) | -3.10 (-3.67, -2.44) | **0.002** | **0.063** | **0.85 [0.69-0.97]** |  |  |
| CTLA4 | -0.40 (-1.29, 0.14) | -0.87 (-1.53, -0.43) | 0.3 | 0.5 | 0.61 [0.27–0.92] |  |  |
| CX3CR1 | -2.67 (-4.56, -2.01) | -1.89 (-2.38, -1.52) | 0.10 | 0.3 | 0.69 [0.40-0.92] |  |  |
| GNLY | -0.23 (-3.23, 0.74) | 0.32 (-0.53, 1.27) | 0.2 | 0.4 | 0.65 [0.39-0.89] |  |  |
| IFNɣ | -4.63 (-6.32, -2.85) | -3.27 (-4.18, -2.58) | 0.3 | 0.4 | 0.63 [0.29-0.91] |  |  |
| IL10 | -11.84 (-13.01, -11.48) | -13.92 (-15.48, -12.63) | **0.008** | **0.074** | **0.80 [0.63-0.95]** |  |  |
| IL1R2 | 3.43 (2.99, 5.61) | 3.00 (2.24, 3.46) | **0.050** | 0.2 | **0.72 [0.52-0.91]** |  |  |
| IL1RN | -4.91 (-5.04, -4.65) | -4.88 (-5.35, -4.09) | 0.9 | 0.9 | 0.52 [0.35–0.71] |  |  |
| IL7R | -2.82 (-3.81, -0.58) | -0.51 (-1.33, 0.09) | 0.11 | 0.3 | 0.68 [0.34-0.97] |  |  |
| IP10 | -7.57 (-9.13, -5.76) | -6.20 (-7.68, -5.06) | 0.2 | 0.4 | 0.64 [0.39-0.88] |  |  |
| MDC1 | -0.36 (-1.69, -0.32) | -1.04 (-1.43, -0.67) | 0.5 | 0.7 | 0.57 [0.24–0.92] |  |  |
| OAS2 | -4.56 (-5.19, -3.62) | -4.29 (-4.86, -3.68) | 0.7 | 0.8 | 0.54 [0.26–0.81] |  |  |
| S100A9 | -0.86 (-2.01, 0.12) | -2.18 (-2.90, -1.59) | **0.01** | **0.090** | **0.78 [0.61-0.95]** |  |  |
| TAP2 | -0.97 (-2.01, 0.12) | -0.44 (-0.81, 0.01) | 0.13 | 0.3 | 0.67 [0.37-0.92] |  |  |
| TDRD9 | -1.75 (-2.88, -0.03) | -2.11 (-2.97, -1.41) | 0.5 | 0.6 | 0.58 [0.27–0.87] |  |  |
| TNF | -0.22 (-0.43, 0.05) | -0.41 (-0.84, -0.09) | 0.3 | 0.4 | 0.62 [0.36-0.86] |  |  |
| ZAP70 | 0.88 (0.14, 1.97) | 1.59 (0.84, 2.21) | 0.3 | 0.4 | 0.62 [0.36-0.86] |  |  |
| For each transcriptomic marker, expression levels measured before liver transplantation are presented according to 1-year survival status as median [interquartile range] in survivors and non-survivors. Group comparisons were performed using Wilcoxon rank-sum tests. False discovery rate (FDR) correction was applied for multiple testing. Discriminatory performance for 1-year mortality prediction was assessed using receiver operating characteristic analyses and is reported as area under the curve (AUC) with 95% confidence intervals. Statistically significant p-values and q-values are shown in bold. AUC values are shown in bold when the 95% confidence interval does not include 0.50. ¹Wilcoxon rank-sum test (significant if p<0.05). ²False discovery rate correction for multiple testing (significant if q<0.10). ^3^Area Under the Curve. | | | | | | |  |
